# Functional impairment profiles and long-term care implications among community health service-managed older adults with disability in China: a latent profile and network analysis study

**DOI:** 10.3389/fpubh.2026.1885713

**Published:** 2026-08-31

**Authors:** Ruishan Liu, Yiheng Chen, Ting Niu, Dingyi Zhang, Yuelin Li, Dan Yang, Yulin Lu

**Affiliations:** 1 School of Nursing, Kunming Medical University, Kunming, China; 2 Jinma Subdistrict Health Service Center, Guandu District, Kunming, China

**Keywords:** functional impairment, latent profile analysis, living arrangement, long-term care, network analysis, older adults with disability

## Abstract

**Objective:**

To identify functional impairment profiles among older adults with disability managed within a community health service catchment in China and examine classification-adjusted associations between living arrangement and profile membership.

**Methods:**

This cross-sectional study included 361 older adults with disability from 13 communities in Kunming, China, surveyed from January to March 2026. Functional ability was assessed using the Specification for Ability Assessment of Older Adults (GB/T 42195–2022). Latent profile analysis used self-care ability, basic mobility, and mental state as indicators; a four-domain model including sensory perception and social participation was tested for sensitivity. Sequential R3STEP models examined living arrangement while accounting for classification uncertainty and progressively adjusting for demographic, socioeconomic, family-resource, and health-related variables. Exploratory network analysis compared the four functional domains between living-arrangement groups.

**Results:**

Four profiles were identified: severe multidomain impairment (28.3%), impaired mental state with relatively preserved physical function (12.7%), moderate functional impairment (53.5%), and relatively preserved function (5.5%). The four-domain model showed 92.5% classification agreement (Cohen's κ = 0.882). In the fully adjusted R3STEP model, compared with institutional living, home living was associated with greater odds of membership in the moderate functional impairment profile rather than the severe multidomain impairment profile (aOR = 7.046, 95% CI: 2.827–17.562; *P* < 0.001) and the relatively preserved function profile (aOR = 15.408, 95% CI: 2.652–89.528; *P* = 0.002), although the latter estimate was imprecise. No significant association was found for the impaired mental state with relatively preserved physical function profile (aOR = 0.570, 95% CI: 0.131–2.476; *P* = 0.453). Estimates were consistent across sequential models. Network analysis showed differences in overall structure and global expected influence, but not global strength; only the self-care ability–mental state edge differed after Holm correction.

**Conclusion:**

Functional impairment was heterogeneous. Home living was associated with membership in the moderate functional impairment and relatively preserved function profiles after adjustment for measured covariates. These cross-sectional associations do not establish causal effects because residual confounding and care-selection effects remain possible. Profile-based assessment may complement existing grading by identifying differentiated care needs.

## 1 Introduction

Disability in later life is a major public health and long-term care challenge in rapidly aging societies. Older adults with disability may experience difficulties in essential activities of daily living, including dressing, eating, bathing, toileting, indoor mobility, and shopping (1). With the continuous intensification of population aging, the long-term care of older adults with disability has become a major focus in the development of health and long-term care service systems worldwide. A cross-national analysis of World Health Survey data found that 33.3% of 53,447 adults aged 50 years and older had disability (2). By the end of 2025, China had 323.38 million people aged 60 years and older, accounting for 23.0% of the national population (3). Research forecasts show that the number of older adults with disability or partial functional dependence in China will reach 76.11 million by 2030 and increase to 120 million by 2050 (4). These trends indicate that care needs related to aging and disability are no longer merely clinical or familial issues, but increasingly severe challenges for community health services, long-term care systems, and the allocation of public health resources.

In recent years, China has introduced several policies specifically addressing the assessment, care, and long-term support needs of older adults with disability. The Notice on Conducting Nursing Needs Assessment and Standardizing Services for Older Adults required nursing needs to be evaluated according to functional ability and geriatric syndromes and linked the assessment results to graded nursing services (5). The Guiding Opinions on Expanding the Pilot Program of Long-term Care Insurance prioritized eligible older adults with disability, required disability assessment for benefit eligibility, and encouraged differentiated coverage according to care level and service setting, including home- and community-based care (6). The Notice on Comprehensively Strengthening Health Services for Older Adults further called for health assessment and services for older adults with disability and for an integrated health-care model extending from professional institutions to communities and homes (7). Together, these policies emphasize standardized assessment, differentiated services, and continuity of care across home, community, and institutional settings for older adults with disability.

At the service level, however, implementing these policy goals requires more than determining whether an older person is disabled or assigning a single disability grade. Community health services and long-term care providers must also determine which functional domains are impaired, which abilities remain, and where further assessment or referral may be needed. Older adults with the same overall grade may differ substantially in mobility, mental state, sensory access, communication, and participation. A domain-pattern perspective may therefore provide a clinically and administratively useful second layer of information, while the overall grade continues to support eligibility determination and broad risk stratification.

In terms of disability evaluation, GB/T 42195–2022, the Specification for Ability Assessment of Older Adults, provides a nationally standardized framework covering self-care ability, basic mobility, mental state, and sensory perception and social participation (8). The standard was introduced in 2022 to provide a unified and operational framework for assessing older adults' abilities and supporting the classification and provision of care services. Its multidimensional structure is suitable for comprehensive assessment, whereas routine interpretation based primarily on a total score or overall grade may obscure distinct combinations of impairment within the same broad disability level. Large-scale Chinese aging cohorts, including CHARLS, CLHLS, and CLASS, contain well-established measures of activities of daily living, instrumental activities of daily living, cognition, sensory function, mobility, socioeconomic conditions, and family resources (9–11). These measures substantially overlap with the constructs assessed by GB/T 42195–2022 and provide important advantages in population coverage, longitudinal follow-up, and covariate availability. However, the commonly used public waves of these cohorts were either completed before the national standard was introduced or were based on cohort-specific assessment instruments. For example, the latest widely available CHARLS national follow-up wave was conducted in 2020, while the most recent CLHLS wave commonly available for research was conducted in 2018. Consequently, these datasets were not designed to administer GB/T 42195–2022 or to generate its four standardized domain scores. The present study therefore collected primary data after implementation of the national standard and assessed home-living and institutional-living older adults within the same community health service catchment using an identical assessment procedure. This approach provided contemporaneous evidence on functional patterns defined directly under GB/T 42195–2022 and reflected the current local service context. Nevertheless, the present data do not replace the broader representativeness, longitudinal information, or extensive contextual variables available in national cohorts. The two types of data should be regarded as complementary sources of evidence. In the present analysis, available demographic, socioeconomic, family-resource, and health-related characteristics were included as covariates, while the possibility of residual confounding was explicitly acknowledged.

Latent profile analysis (LPA) is a person-centered method that identifies unobserved subgroups based on patterns across continuous indicators (12). Previous studies have demonstrated heterogeneity in disability patterns, support needs, and care preferences among older adults (13–15), indicating that functional impairment may not be adequately represented by a single continuum from mild to severe. However, limited evidence is available on functional profiles among older adults with disability who are already managed within community health service systems, particularly when home-living and institutional-living individuals are assessed contemporaneously using the same national standard. It also remains uncertain whether profiles derived from the core physical and mental domains remain stable when sensory perception and social participation are included, and whether living arrangement remains associated with profile membership after uncertainty in latent profile classification and measured demographic, socioeconomic, family-resource, and health-related differences are taken into account.

Living arrangement is an important contextual characteristic because home and institutional settings may differ in care organization, environmental familiarity, autonomy, informal caregiving, and opportunities for social interaction. Previous longitudinal research has reported associations between living arrangement and subsequent activities-of-daily-living disability (16). However, residence in an institutional care setting is not randomly assigned. The decision to remain at home or enter an institution may be influenced by pre-existing functional status, educational and economic circumstances, marital status, availability of children or other family caregivers, health burden, and access to formal care. Many of these factors may also be independently associated with functional impairment. Consequently, an observed association between living arrangement and functional profile membership may reflect both differences between care settings and selection into those settings.

Sensory perception and social participation are also relevant to functional status. Social participation has been associated with functional decline, incident disability, and mortality among older adults (17–19), whereas visual and hearing impairment may restrict communication, environmental adaptation, functional independence, and cognitive engagement (20, 21). In the present study, living arrangement was therefore examined as the primary external characteristic associated with profile membership, with adjustment for available demographic, socioeconomic, family-resource, and health-related variables. Sensory perception and social participation were examined separately in the sensitivity, network, and exploratory statistical indirect-association analyses. Because the study was cross-sectional and several determinants of care selection were not measured, none of these associations were interpreted as evidence that one living arrangement caused better or worse functional outcomes.

Functional domains may also be interrelated rather than declining independently. LPA describes heterogeneity in combinations of impairment, whereas network analysis offers a complementary description of conditional associations among measured domains. Network methods can estimate the relative pattern and strength of these associations and assess whether their configuration differs between groups (22, 23). In the present study, such analyses were used only as exploratory and supplementary tools and not to identify causal mechanisms or intervention targets.

Accordingly, this study aimed to identify functional impairment profiles among older adults with disability managed within one community health service catchment, including both home-living and institutional-living participants assessed using GB/T 42195-2022. We first conducted a primary LPA using self-care ability, basic mobility, and mental state and evaluated competing profile solutions. A four-domain LPA including sensory perception and social participation was performed as a sensitivity analysis. We then used sequential R3STEP models to examine classification-adjusted associations between living arrangement and profile membership under progressively expanded covariate adjustment. Statistical indirect associations were examined cautiously, and an exploratory four-node network analysis compared conditional domain relationships across living arrangements. Together, these analyses were intended to describe functional heterogeneity and generate service-level hypotheses for differentiated assessment and care coordination rather than to establish causal pathways or nationally generalizable management rules. The specific contribution of this study was to identify distinct patterns of functional impairment among home-living and institutional-living older adults assessed with GB/T 42195–2022 within the same community health service catchment. We also accounted for uncertainty in profile classification and examined whether the identified profile structure remained stable in sensitivity analyses.

## 2 Materials and methods

### 2.1 Study design and participants

A cross-sectional survey was conducted from January to March 2026 in the service catchment of a first-class community health service center covering 13 communities in Kunming, China. The target population was older adults with disability included in health management by the community health service center. Two living-arrangement groups were included: older adults living at home and those living in care institutions located within the same catchment. In the local service system, the community health service center undertakes health assessment, follow-up, referral, and care coordination for eligible older adults in both settings. Community health service management therefore defined the sampling frame, whereas living arrangement was analyzed as a participant characteristic.

**Figure 1:**
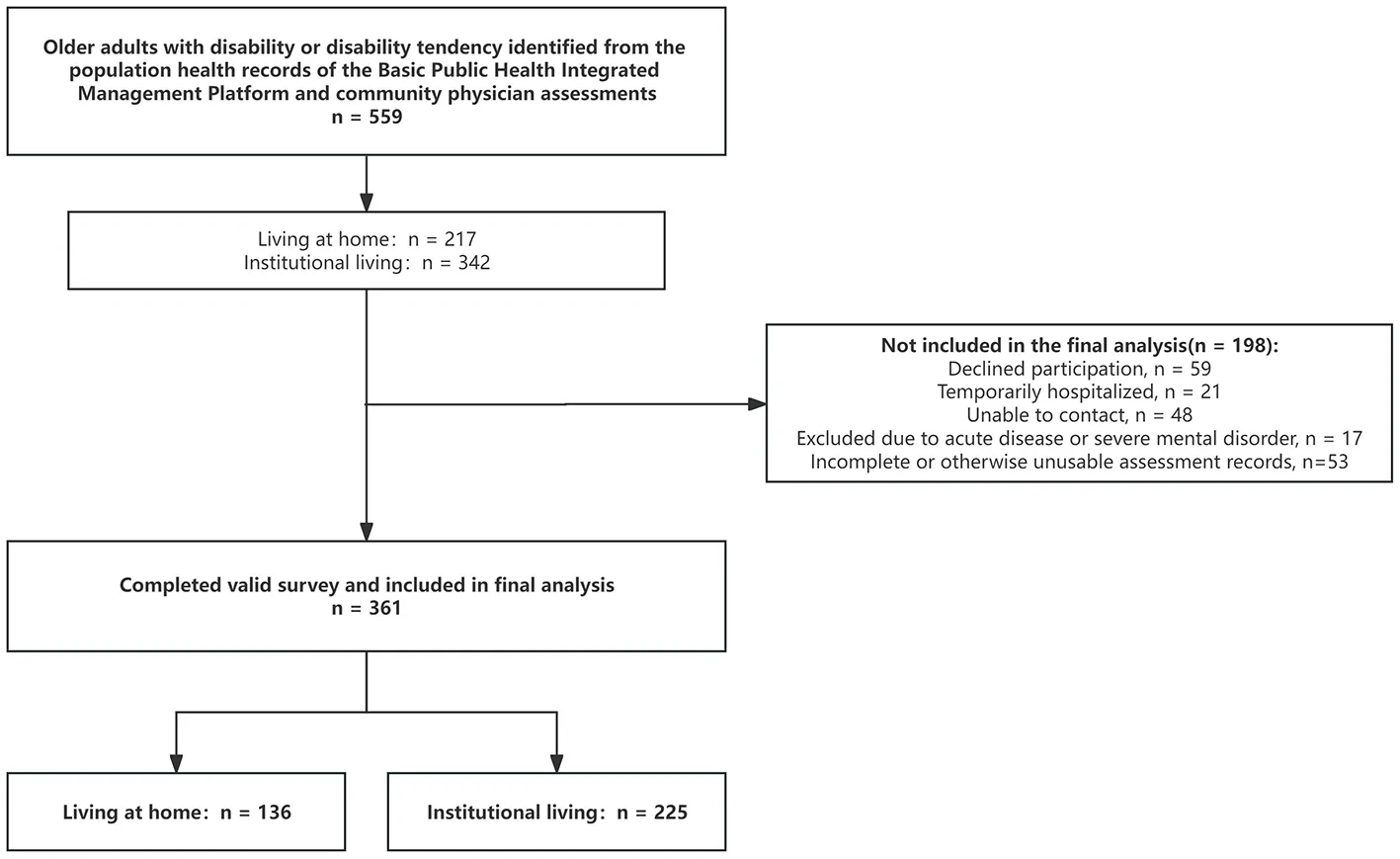
Flow diagram of participant recruitment and inclusion.

The service catchment was selected purposively. Within this catchment, the population health records of the Basic Public Health Integrated Management Platform and community physician assessments identified 559 older adults with disability or disability tendency, including 217 living at home and 342 living in institutions. Recruitment was attempted for all 559 individuals. Of these, 198 were not included in the final analysis: 59 declined participation, 21 were temporarily hospitalized, 48 could not be contacted, 17 were excluded because of acute disease or severe mental disorder, and 53 had incomplete or otherwise unusable assessment records because of missing required general information, duplicate assessment entries, or inability to complete the face-to-face assessment. Among the 511 individuals for whom contact was established, 59 declined participation, corresponding to a refusal rate of 11.5%. The final analytic sample comprised 361 participants, including 136 living at home and 225 living in institutions (Figure 1).

Inclusion criteria were: (i) age ≥60 years; (ii) meeting the Ministry of Civil Affairs assessment criteria for disability or disability tendency, ranging from mild impairment to complete loss of ability (8); and (iii) provision of informed consent and ability to participate in the face-to-face assessment. When necessary, caregivers or family members supplemented background and health-related information. Exclusion criteria were acute disease, severe mental disorder, or another condition identified before assessment that made participation impossible. Using Kendall's rule of 5–10 participants per variable (24), the 36 questionnaire and assessment items indicated a target sample of 200–400 after allowing for 10% attrition; the final sample of 361 met this requirement. The study was approved by the Ethics Committee of Kunming Medical University (KMMU2026MEC009). Written informed consent was obtained from participants or their legal guardians/close relatives.

### 2.2 Measures

Data were collected through assessor-administered, face-to-face assessments rather than self-administered questionnaires. General and health-related information was obtained from identity information, health records, the participant, a caregiver or family member, and available diagnostic documentation, as appropriate. Information obtained through participant or family report was cross-checked against available health records or diagnostic documentation when clinical confirmation was required. Trained healthcare assessors completed and scored the 26 ability-assessment items in accordance with GB/T 42195–2022. Assessment records were excluded from the final analysis if required general-information variables were missing, duplicate assessment entries were identified, or the face-to-face assessment could not be completed. No item-level prorating or imputation was performed.

**(i) General and health-related information:** Sex and age were obtained from identity information and health records and cross-checked with participant or family report. Living arrangement was determined according to the participant's usual place of residence at the time of assessment. Consistent with the Chinese statistical definition of usual residence (25), participants who had lived at home or in a care institution within the service catchment for at least 6 months were classified according to their usual living arrangement. Individuals who were temporarily hospitalized during recruitment were not assessed.

General information included sex, age, marital status, living arrangement, educational attainment, monthly household income, number of children, primary caregiver, and medical insurance type. Health-related information included chronic disease status and geriatric syndrome burden. Chronic disease status was coded as present when at least one chronic disease was diagnosed by a community physician or documented by a Grade III Class A hospital. Participant or family reports were verified against available clinical records before coding when clinical confirmation was required. Geriatric syndrome burden was based on conditions documented in health records or confirmed by a community physician during the face-to-face assessment.

For descriptive analyses, age was categorized as < 70, 70–79, 80–89, or ≥90 years; marital status as never married, married, divorced, or widowed; living arrangement as institutional living, home living with family, or living alone; educational attainment as no formal schooling, primary school, junior high school, senior high school/technical secondary school, or college and above; monthly household income as < RMB 3,000, RMB 3,000–5,999, RMB 6,000–9,999, or ≥RMB 10,000; number of children as 0, 1, 2, or ≥3; medical insurance as employee medical insurance, urban–rural resident medical insurance, or no medical insurance; and geriatric syndrome burden as 0, 1–2, or ≥3 syndromes.

For the R3STEP analyses, participants living at home with family and those living alone were combined into the home-living group and compared with participants living in institutions. Age was modeled per 10-year increase, and marital status was dichotomized as currently married vs. not currently married. Educational attainment was represented by two indicator variables—primary school and junior high school or above—with no formal schooling as the reference. Monthly household income was represented by two indicator variables—RMB 3,000–5,999 and ≥RMB 6,000—with < RMB 3,000 as the reference. Number of children and geriatric syndrome burden were entered as ordinal variables according to the categories defined above. Medical insurance type was represented by two indicator variables, with no medical insurance as the reference. Primary caregiver was reported descriptively but was not included as a conventional covariate because it was structurally dependent on living arrangement, particularly among institutional-living participants.

(ii) **The specification for ability assessment of older adults** comprises 26 items across four domains: self-care ability, basic mobility, mental state, and sensory perception and social participation (8). Self-care ability includes eating, grooming, bathing, dressing, bowel and bladder control, toileting, bed-chair transfer, and walking on level ground. Basic mobility includes position changes in bed, maintaining sitting and standing positions, and stair climbing. Mental state includes orientation, memory, comprehension, expression, aggressive behavior, depressive symptoms, and level of consciousness. Sensory perception and social participation includes vision, hearing, handling daily affairs, transportation use, and social interaction. Items were scored according to the national specification, with higher scores indicating better ability. At the item level, the 26-item assessment showed high overall internal consistency in the present sample (Cronbach's α = 0.967). The Kaiser–Meyer–Olkin value was 0.947, and Bartlett's test of sphericity was significant (*P* < 0.001), indicating that the inter-item correlation matrix was suitable for factor-related analyses. These statistics were not interpreted as definitive evidence of construct validity or as confirmation of the four-domain structure.

For the primary LPA, self-care ability, basic mobility, and mental state were used as manifest indicators because they represented the core physical and mental functional patterns used to define profiles. Domain scores were linearly transformed to a 0–100 scale to permit comparison across domains, with higher scores indicating better functional ability.

The sensory perception and social participation domain was not included in the primary profile definition. Its original score ranged from 0 to 15, with higher scores indicating better sensory perception and greater social participation. The original score was retained as an external continuous variable for descriptive comparisons and the exploratory statistical indirect-association analysis. It was not included as a covariate in the primary R3STEP analysis, which focused on the classification-adjusted association between living arrangement and profile membership. This analytical separation was intended to avoid using the same domain both to define the primary latent profiles and as a principal explanatory variable in the R3STEP model. However, because all four domains were derived from the same assessment instrument, the separation did not eliminate conceptual overlap or common-method bias. To examine whether the identified profile structure was sensitive to inclusion of the fourth domain, a separate LPA incorporating self-care ability, basic mobility, mental state, and sensory perception and social participation was conducted.

For the exploratory network analysis, all four domain scores were linearly transformed to a 0–100 scale and entered as four network nodes. This domain-level specification was consistent with the structure of GB/T 42195–2022 and provided a supplementary description of conditional associations among the measured functional domains. The network analysis was conducted separately from the primary LPA and did not contribute to the definition or assignment of the latent profiles.

### 2.3 Statistical analysis

SPSS version 26.0 was used for descriptive statistics and univariate analyses. Categorical variables were summarized as *n* (%) and compared using Pearson's chi-square test or the Fisher-Freeman-Halton exact test, as appropriate. Sensory perception and social participation scores were non-normally distributed across most profiles and were summarized as median (P25, P75) and compared using the Kruskal-Wallis *H* test. Bonferroni-adjusted pairwise comparisons followed a significant overall test. All tests were two-sided, with *P* < 0.05 considered statistically significant.

Mplus version 8.3 was used for LPA based on the 0–100 scores of self-care ability, basic mobility, and mental state. One- to five-profile models were estimated with 1,000 random starts and 250 final-stage optimizations. Within-profile covariances were fixed at zero and residual variances were constrained to be equal across profiles. Model selection considered the Akaike information criterion (AIC), Bayesian information criterion (BIC), sample size-adjusted BIC (aBIC), entropy, Lo-Mendell-Rubin adjusted likelihood ratio test (LMRT), bootstrap likelihood ratio test (BLRT), class sizes, average posterior probabilities, parsimony, and substantive interpretability. No absolute minimum class-size threshold was applied; smaller profiles were evaluated in conjunction with model fit, classification quality, profile separation, total sample size, and substantive interpretability (12, 26). Three- and four-profile assignments were cross-tabulated after alignment by domain-score patterns; similarity was quantified using the adjusted Rand index, and overall agreement and Cohen's kappa were calculated after collapsing the additional relatively preserved profile for sensitivity purposes. A separate one- to five-profile sensitivity LPA included all four domains under the same model constraints. Its retained four-profile assignments were compared with those from the primary three-domain model using cross-classification, adjusted Rand index, overall agreement, and Cohen's kappa.

Associations between living arrangement and latent profile membership were estimated using the auxiliary R3STEP procedure in Mplus, which accounts for uncertainty in latent profile classification while relating covariates to profile membership. The severe multidomain impairment profile was specified as the reference outcome category. Participants living at home with family and those living alone were combined into the home-living group, and institutional living was used as the reference exposure category. Three sequential R3STEP models were estimated to examine the stability of the association after progressive covariate adjustment. Model 0 included living arrangement only. Model 1 additionally adjusted for sex, age, current marital status, educational attainment, monthly household income, and medical insurance type. Model 2 further adjusted for number of children, chronic disease status, and geriatric syndrome burden. Model 2 was treated as the fully adjusted primary model. Sensory perception and social participation were not included in these R3STEP models because this domain was examined separately in the sensitivity, network, and exploratory statistical indirect-association analyses. Age was modeled per 10-year increase. Current marital status was coded as currently married vs. not currently married. Educational attainment and monthly household income were represented using indicator variables, whereas number of children and geriatric syndrome burden were entered as ordinal variables according to the categories described above. Primary caregiver was not included in the multivariable model because it was structurally dependent on living arrangement, particularly among institutional-living participants. Estimates for the relatively preserved function profile were interpreted cautiously because this profile contained only 20 participants.

Exploratory network analysis was conducted in *R* version 4.5.2. Gaussian graphical models were estimated for the full sample and separately for the home-living and institutional-living groups using EBICglasso with an EBIC tuning parameter of 0.50 and correlations estimated using cor_auto. Strength and expected influence were reported descriptively. Edge-weight accuracy was assessed using 2,000 nonparametric bootstrap samples, and centrality stability using 2,000 case-dropping bootstrap samples and the correlation-stability coefficient. The Network Comparison Test used 5,000 permutations to compare overall network structure, global strength, and global expected influence. When the overall structure test was significant, Holm-adjusted *post hoc* edge comparisons were performed. Given the four-node specification, all network findings were considered exploratory and supplementary.

Statistical indirect associations were examined in R, with living arrangement as the independent variable, sensory perception and social participation as the intermediate variable, and most likely profile membership as the multicategory outcome. Living arrangement was coded as 0 for institutional living and 1 for home living, and the severe multidomain impairment profile was specified as the reference outcome category. The intermediate-variable model was estimated using multiple linear regression, and the outcome model was estimated using multinomial logistic regression. Both models were adjusted for sex, age, current marital status, chronic disease status, and medical insurance type. Product-of-coefficients estimates and percentile 95% confidence intervals were obtained using 5,000 nonparametric bootstrap samples. Because all variables were measured cross-sectionally and the outcome was based on most likely profile assignment, the estimates were interpreted as exploratory statistical indirect associations rather than causal mediation effects. In addition, because multinomial logistic regression is non-linear, the direct, indirect, and total association coefficients were not interpreted as an exact additive decomposition.

## 3 Results

### 3.1 Identification of functional impairment profiles

After comparing one- to five-profile models, the four-profile solution was retained. Relative to the three-profile model, it had lower AIC, BIC, and aBIC values, higher entropy (0.859), significant LMRT and BLRT results, and diagonal average posterior probabilities of 0.872–0.945. Although the five-profile solution further reduced the information criteria and increased entropy, it produced a class of 16 participants (4.4%) and a diagonal posterior probability of 0.747 for one class, with limited additional substantive differentiation. Considering the improved model fit, acceptable classification quality, distinct profile pattern, class size, parsimony, and substantive interpretability, the four-profile model was selected (Table 1). Class sizes for all competing models are provided in Supplementary Table S1.

**Table 1:** Fit indices of latent profile analysis models.

Profile	AIC	BIC	aBIC	Entropy	***P***		Estimated class probability	Average posterior probabilities
					LMRT	BLRT		
1	10,052.363	10,075.696	10,056.661					
2	9,909.941	9,948.830	9,917.105	0.718	<0.001^*^	<0.001^*^	0.33795/0.66205	0.906/0.928
3	9,781.321	9,835.766	9,791.350	0.847	0.0489^*^	<0.001^*^	0.31856/0.11911/0.56233	0.910/0.924/0.944
**4**	**9,732.768**	**9,802.768**	**9,745.662**	**0.859**	**0.0001**	**<0.001** ^*^	**0.28255/0.12742/0.53463/ 0.05540**	**0.914/0.945/0.926/ 0.872**
5	9,677.996	9,763.551	9,693.756	0.878	0.0028	<0.001^*^	0.04432/0.51247/0.28809/ 0.09695/0.05817	0.915/0.925/0.923/0.941/ 0.747

AIC, Akaike information criterion; BIC, Bayesian information criterion; aBIC, sample size-adjusted Bayesian information criterion; LMRT, Lo-Mendell-Rubin adjusted likelihood ratio test; BLRT, bootstrap likelihood ratio test. Estimated class probabilities are model-estimated proportions; average posterior probabilities are diagonal classification probabilities. The four-profile solution was retained after considering statistical fit, classification quality, class size, parsimony, and substantive interpretability.

^*^P < 0.05. Bold values indicate the retained four-profile solution.

**Figure 2:**
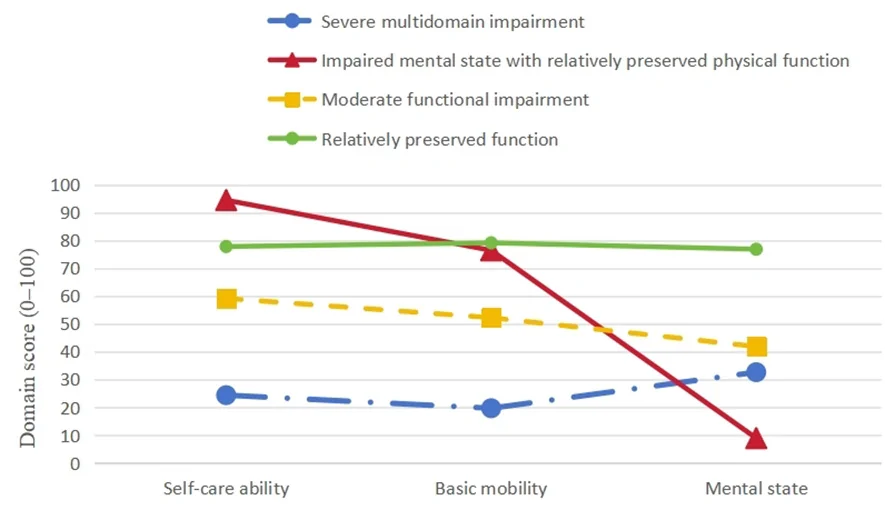
Functional impairment profiles across three core functional domains. Points and lines represent model-estimated profile-specific means. Domain scores were linearly transformed to a 0–100 scale, with higher scores indicating better functional ability.

Profile 1 was labeled severe multidomain impairment because model-estimated mean scores were low for self-care ability (24.4), basic mobility (19.7), and mental state (32.6). Profile 2, impaired mental state with relatively preserved physical function, showed high self-care ability (94.4) and basic mobility (76.3) but markedly low mental state (8.9). Profile 3, moderate functional impairment, had intermediate scores across self-care ability (59.1), basic mobility (52.2), and mental state (41.8). Profile 4, relatively preserved function, had comparatively high scores across all three domains (77.7, 79.1, and 76.7, respectively). Based on most likely membership, the profiles included 102 (28.3%), 46 (12.7%), 193 (53.5%), and 20 (5.5%) participants, respectively (Figure 2; Supplementary Table S2).

The three-profile vs. four-profile sensitivity analysis indicated that the principal structure was retained. Of 115 participants in the three-profile severe multidomain impairment group, 102 (88.7%) remained in the corresponding four-profile group. All 43 participants in the impaired mental state with relatively preserved physical function group retained the corresponding classification. Of 203 participants in the three-profile moderate functional impairment group, 180 (88.7%) remained in that profile and 20 (9.9%) were differentiated into the relatively preserved function profile. The adjusted Rand index was 0.746. After collapsing the moderate and relatively preserved profiles in the four-profile solution, overall agreement was 95.6% and Cohen's kappa was 0.921 (Supplementary Table S3 and Supplementary Figure S1).

In the four-domain sensitivity analysis, the retained four-profile solution reproduced the substantive patterns identified in the primary three-domain model. The four profiles comprised 104 participants (28.8%) with severe multidomain impairment, 46 (12.7%) with impaired mental state and relatively preserved physical function, 178 (49.3%) with moderate functional impairment, and 33 (9.1%) with relatively preserved function. Classification quality was acceptable, with an entropy of 0.843 and diagonal average posterior probabilities ranging from 0.900 to 0.951. Although the LMRT comparing the four- and three-profile solutions was not statistically significant (*P* = 0.156), the four-profile solution was retained for sensitivity comparison because it yielded substantively interpretable profiles without a class smaller than 5%, whereas the five-profile solution included a class comprising only 4.7% of the sample. Cross-classification with the primary three-domain model showed 92.5% overall agreement, an adjusted Rand index of 0.799, and a Cohen's κ of 0.882 (Supplementary Tables S4–S6).

### 3.2 Factors associated with functional impairment profile membership

Among the 361 participants, 208 (57.6%) were women, 219 (60.7%) were aged ≥80 years, and 172 (47.6%) were married. Regarding living arrangement, 225 participants (62.3%) lived in institutions, 111 (30.7%) lived at home with family members, and 25 (6.9%) lived alone. Overall, 211 participants (58.4%) had no formal schooling or primary-school education, 301 (83.4%) had a monthly household income below RMB 6,000, and 218 (60.4%) had at least two children. An institutional care worker was the primary caregiver for 208 participants (57.6%). In addition, 235 participants (65.1%) had at least one chronic disease, and 312 (86.4%) had at least one recorded geriatric syndrome. Employee medical insurance, urban–rural resident medical insurance, and no medical insurance accounted for 49.6%, 34.3%, and 16.1% of the sample, respectively. Participant characteristics and the unadjusted distribution of functional impairment profiles according to living arrangement are presented in Supplementary Table S7.

Univariate comparisons showed statistically significant differences across the four profiles in marital status (Fisher–Freeman–Halton exact test, Monte Carlo *P* = 0.028), living arrangement (χ^2^ = 60.616, *P* < 0.001), primary caregiver (Fisher–Freeman–Halton exact test, Monte Carlo *P* < 0.001), geriatric syndrome count (χ^2^ = 14.002, *P* = 0.030), and sensory perception and social participation score (H = 89.560, *P* < 0.001). No statistically significant differences were observed in sex (*P* = 0.212), age group (P = 0.497), educational attainment (Monte Carlo *P* = 0.687), monthly household income (Monte Carlo *P* = 0.698), number of children (*P* = 0.675), medical insurance type (*P* = 0.498), or chronic disease status (*P* = 1.000) (Table 2).

**Table 2:** Univariate comparisons of participant characteristics across the four functional impairment profiles (*n* = 361).

Variable	Severe multidomain impairment (*n* = 102)	Impaired mental state with relatively preserved physical function (*n* = 46)	Moderate functional impairment (*n* = 193)	Relatively preserved function (*n* = 20)	Test statistic	*P* value
Sex						
Male	36 (35.3%)	17 (37.0%)	91 (47.2%)	9 (45.0%)	4.506^a^	0.212
Female	66 (64.7%)	29 (63.0%)	102 (52.8%)	11 (55.0%)		
Age						
< 70	6 (5.9%)	4 (8.7%)	15 (7.8%)	3 (15.0%)	8.376^a^	0.497
70–79	30 (29.4%)	12 (26.1%)	63 (32.6%)	9 (45.0%)		
80–89	40 (39.2%)	17 (37.0%)	77 (39.9%)	7 (35.0%)		
≥90	26 (25.5%)	13 (28.3%)	38 (19.7%)	1 (5.0%)		
Marital status						
Never married	2 (2.0%)	4 (8.7%)	3 (1.6%)	0 (0.0%)	17.038^b^	0.028
Married	37 (36.3%)	20 (43.5%)	105 (54.4%)	10 (50.0%)		
Divorced	5 (4.9%)	1 (2.2%)	7 (3.6%)	2 (10.0%)		
Widowed	58 (56.9%)	21 (45.7%)	78 (40.4%)	8 (40.0%)		
Living arrangement						
Institutional living	84 (82.4%)	41 (89.1%)	93 (48.2%)	7 (35.0%)	60.616^a^	< 0.001
Home living with family	12 (11.8%)	4 (8.7%)	82 (42.5%)	13 (65.0%)		
Living alone	6 (5.9%)	1 (2.2%)	18 (9.3%)	0 (0.0%)		
Monthly household income, RMB						
< 3,000	41 (40.2%)	13 (28.3%)	76 (39.4%)	7 (35.0%)	6.339^b^	0.698
3,000–5,999	48 (47.1%)	25 (54.3%)	83 (43.0%)	8 (40.0%)		
6,000–9,999	7 (6.9%)	6 (13.0%)	23 (11.9%)	3 (15.0%)		
≥10,000	6 (5.9%)	2 (4.3%)	11 (5.7%)	2 (10.0%)		
Educational attainment						
No formal schooling	24 (23.5%)	14 (30.4%)	55 (28.5%)	8 (40.0%)	8.947^b^	0.687
Primary school	38 (37.3%)	11 (23.9%)	54 (28.0%)	7 (35.0%)		
Junior high school	23 (22.5%)	13 (28.3%)	53 (27.5%)	3 (15.0%)		
Senior high school/technical secondary school	11 (10.8%)	6 (13.0%)	26 (13.5%)	2 (10.0%)		
College or above	6 (5.9%)	2 (4.3%)	5 (2.6%)	0 (0.0%)		
Number of children						
0	8 (7.8%)	3 (6.5%)	22 (11.4%)	1 (5.0%)	6.640^a^	0.675
1	27 (26.5%)	18 (39.1%)	59 (30.6%)	5 (25.0%)		
2	42 (41.2%)	17 (37.0%)	73 (37.8%)	7 (35.0%)		
≥3	25 (24.5%)	8 (17.4%)	39 (20.2%)	7 (35.0%)		
Primary caregiver						
Spouse	6 (5.9%)	2 (4.3%)	33 (17.1%)	5 (25.0%)	50.985^b^	< 0.001
Children or children-in-law	16 (15.7%)	4 (8.7%)	55 (28.5%)	5 (25.0%)		
Other relatives	1 (1.0%)	1 (2.2%)	10 (5.2%)	2 (10.0%)		
Institutional care worker	78 (76.5%)	38 (82.6%)	86 (44.6%)	6 (30.0%)		
No regular caregiver	1 (1.0%)	1 (2.2%)	9 (4.7%)	2 (10.0%)		
Medical insurance type						
Employee	49 (48.0%)	25 (54.3%)	97 (50.3%)	8 (40.0%)	5.363^a^	0.498
Urban and rural	36 (35.3%)	13 (28.3%)	64 (33.2%)	11 (55.0%)		
No	17 (16.7%)	8 (17.4%)	32 (16.6%)	1 (5.0%)		
Geriatric syndrome count						
0	11 (10.8%)	1 (2.2%)	34 (17.6%)	3 (15.0%)	14.002^a^	0.030
1–2	66 (64.7%)	29 (63.0%)	126 (65.3%)	11 (55.0%)		
≥3	25 (24.5%)	16 (34.8%)	33 (17.1%)	6 (30.0%)		
Chronic disease						
Yes	66 (64.7%)	30 (65.2%)	126 (65.3%)	13 (65.0%)	0.010^a^	1.000
No	36 (35.3%)	16 (34.8%)	67 (34.7%)	7 (35.0%)		
**Sensory perception and social participation, median (P25, P75)**	4.0 (3.0, 5.0)^A^	5.0 (4.0, 7.0)^AB^	6.0 (5.0, 8.0)^B^	10.0 (9.0, 11.5)^c^	89.560^c^	<0.001

Data are presented as *n* (%) or median (P25, P75).

^a^ Pearson's chi-square test.

^b^ Fisher–Freeman–Halton exact test with *P* values estimated using 10,000 Monte Carlo samples.

^c^ Kruskal–Wallis H test.

For sensory perception and social participation, values that do not share a common uppercase superscript letter differ significantly in Bonferroni-adjusted pairwise comparisons. All *P* values are two-sided.

Classification-adjusted associations between living arrangement and profile membership were examined using the R3STEP procedure, with the severe multidomain impairment profile specified as the reference outcome category. The fully adjusted model included living arrangement, sex, age, current marital status, educational attainment, monthly household income, medical insurance type, number of children, chronic disease status, and geriatric syndrome burden. After adjustment for these measured demographic, socioeconomic, family-resource, and health-related variables, home living was associated with greater odds of membership in the moderate functional impairment profile rather than the severe multidomain impairment profile (aOR = 7.046, 95% CI: 2.827–17.562, *P* < 0.001). Home living was also associated with greater odds of membership in the relatively preserved function profile (aOR = 15.408, 95% CI: 2.652–89.528, *P* = 0.002). However, the latter estimate was imprecise, as indicated by the wide confidence interval and the small size of the relatively preserved function profile (*n* = 20). No statistically significant association was observed between home living and membership in the impaired mental state with relatively preserved physical function profile (aOR = 0.570, 95% CI: 0.131–2.476, *P* = 0.453) (Table 3).

**Table 3:** Classification-adjusted associations between covariates and functional impairment profile membership in the fully adjusted R3STEP model.

Variable	Profile 2 vs. Profile 1, aOR (95% CI)	*P* value	Profile 3 vs. Profile 1, aOR (95% CI)	*P* value	Profile 4 vs. Profile 1, aOR (95% CI)	*P* value
Home living vs. institutional living	0.570 (0.131–2.476)	0.453	**7.046 (2.827–17.562)**	**< 0.001**	**15.408 (2.652–89.528)**	**0.002**
Male vs. female	1.211 (0.536–2.737)	0.645	1.433 (0.700–2.935)	0.325	1.927 (0.410–9.056)	0.406
Age, per 10-year increase	1.082 (0.732–1.598)	0.693	0.979 (0.709–1.353)	0.898	0.703 (0.445–1.109)	0.129
Currently married vs. not currently married	1.565 (0.696–3.519)	0.279	1.421 (0.677–2.981)	0.353	0.926 (0.135–6.351)	0.938
Educational attainment						
Primary school vs. no formal schooling	0.495 (0.173–1.418)	0.190	0.423 (0.176–1.017)	0.055	0.301 (0.071–1.269)	0.102
Junior high school or above vs. no formal schooling	0.849 (0.322–2.241)	0.742	0.849 (0.352–2.048)	0.715	0.210 (0.015–2.976)	0.248
Monthly household income, RMB						
3,000–5,999 vs. < 3,000	1.694 (0.716–4.007)	0.230	1.026 (0.482–2.181)	0.948	1.098 (0.200–6.027)	0.914
≥6,000 vs. < 3,000	1.894 (0.532–6.740)	0.324	1.122 (0.426–2.955)	0.816	2.183 (0.398–11.975)	0.369
Number of children, per category increase	0.774 (0.494–1.211)	0.261	0.770 (0.528–1.122)	0.173	1.277 (0.607–2.689)	0.520
Medical insurance type						
Employee medical insurance vs. no medical insurance	0.943 (0.329–2.706)	0.913	1.114 (0.413–3.003)	0.831	1.911 (0.039–92.971)	0.744
Urban–rural resident medical insurance vs. no medical insurance	0.740 (0.228–2.399)	0.615	0.813 (0.297–2.222)	0.686	4.492 (0.130–154.695)	0.405
Having chronic disease vs. no chronic disease	0.927 (0.402–2.137)	0.859	1.052 (0.522–2.121)	0.887	1.132 (0.142–9.037)	0.907
Geriatric syndrome burden, per category increase	1.722 (0.834–3.555)	0.142	0.610 (0.334–1.113)	0.107	1.417 (0.194–10.363)	0.732

Profile 1, the severe multidomain impairment profile, was used as the reference outcome category. Profile 2 represents the impaired mental state with relatively preserved physical function profile; Profile 3 represents the moderate functional impairment profile; and Profile 4 represents the relatively preserved function profile. Associations were estimated using the R3STEP procedure in Mplus to account for uncertainty in latent profile classification. All covariates shown were entered simultaneously. Age was modeled per 10-year increase. “Not currently married” included never-married, divorced, and widowed participants. Educational attainment was represented by two indicator variables, with no formal schooling as the reference. Monthly household income was represented by two indicator variables, with < RMB 3,000 as the reference. Number of children was coded ordinally as 0, 1, 2, and ≥3, and the aOR represents a one-category increase. Geriatric syndrome burden was coded as 0, 1–2, and ≥3 syndromes, and the aOR represents a one-category increase. *P* values were derived from Wald tests of the corresponding logit coefficients. Estimates for Profile 4 should be interpreted cautiously because this profile contained only 20 participants. aOR, adjusted odds ratio; CI, confidence interval. Bold values indicate statistically significant associations (*P* < 0.05).

The association estimates for living arrangement remained broadly consistent across the sequential R3STEP models. For the moderate functional impairment profile vs. the severe multidomain impairment profile, the OR was 6.918 (95% CI: 3.191–15.000) in Model 0, the aOR was 6.858 (95% CI: 2.832–16.606) in Model 1, and the aOR was 7.046 (95% CI: 2.827–17.562) in Model 2; all three estimates were statistically significant at *P* < 0.001. For the relatively preserved function profile, the corresponding estimates were 12.111 (95% CI: 3.397–43.177), 14.081 (95% CI: 3.111–63.739), and 15.408 (95% CI: 2.652–89.528), respectively. In contrast, the association with the impaired mental state with relatively preserved physical function profile remained statistically nonsignificant in all three models. These findings indicated no material attenuation or reversal in the direction of the living-arrangement estimates after progressive adjustment for the measured covariates (Supplementary Table S8).

None of the other covariates was statistically significantly associated with profile membership in the fully adjusted comparisons with the severe multidomain impairment profile. The association between primary-school education and membership in the moderate functional impairment profile approached, but did not reach, statistical significance (aOR = 0.423, 95% CI: 0.176–1.017, *P* = 0.055) (Table 3). An exploratory R3STEP model that additionally included sensory perception and social participation produced unstable estimates for the smallest profile, consistent with sparse-data separation, and was therefore not interpreted.

### 3.3 Exploratory network analysis of the four functional domains across living-arrangement groups

In the full-sample network, the strongest regularized partial associations were observed between self-care ability and basic mobility (*r* = 0.599) and between mental state and sensory perception and social participation (*r* = 0.582). A positive association was also observed between basic mobility and sensory perception and social participation (r = 0.366). Basic mobility had the highest strength value (1.130), whereas sensory perception and social participation had the highest expected influence value (0.949). Given the exploratory four-node specification, these centrality indices were interpreted descriptively rather than as evidence that any domain represented a causal or intervention target.

In the full-sample network, the correlation-stability coefficients were 0.596 for strength and 0.751 for expected influence. In the institutional-living network, the corresponding coefficients were 0.440 and 0.671, respectively. By contrast, the coefficients were 0.000 for strength and 0.206 for expected influence in the home-living network, indicating insufficient stability for reliable interpretation of node-centrality rankings in this subgroup. Accordingly, subgroup differences in individual centrality rankings were not substantively interpreted.

**Figure 3:**
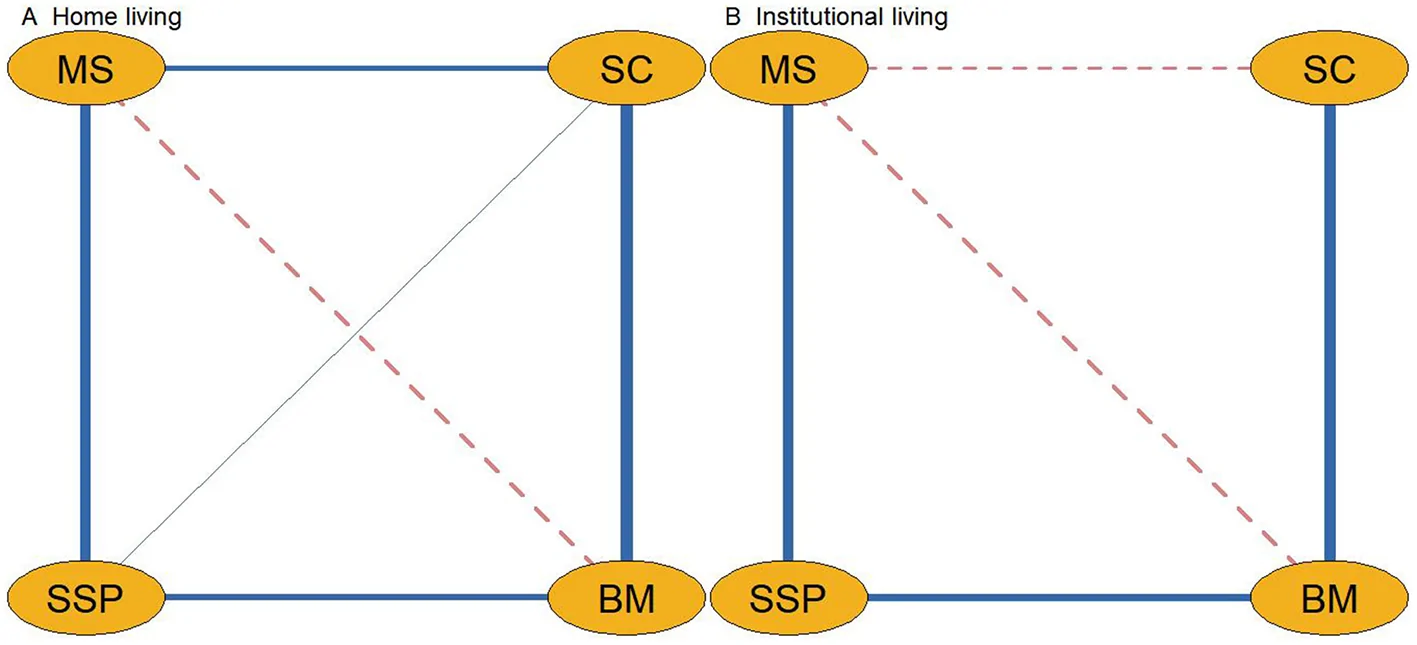
Network structures of the four functional domains in the home-living and institutional-living groups. Panel A represents the home-living group, and Panel B represents the institutional-living group. SC, self-care ability; BM, basic mobility; MS, mental state; SSP, sensory perception and social participation. Solid blue lines indicate positive regularized partial correlations, whereas dashed red lines indicate negative regularized partial correlations. Line thickness represents the absolute magnitude of the edge weight. The two networks were plotted using identical node positions and edge-weight scales. The Network Comparison Test indicated a difference in overall network structure; however, after Holm correction, only the SC–MS edge differed significantly between groups (adjusted *P* = 0.0048).

The Network Comparison Test indicated a statistically significant difference in overall network structure between the home-living and institutional-living groups (*M* = 0.352, *P* = 0.003). Global strength did not differ significantly between the two groups (1.906 vs. 1.685; *S* = 0.221, *P* = 0.493), whereas global expected influence was higher in the home-living network than in the institutional-living network (1.520 vs. 1.109; *S* = 0.411, *P* = 0.005). However, after Holm correction for multiple edge comparisons, only the association between self-care ability and mental state differed significantly between groups (adjusted *P* = 0.0048). This regularized partial association was positive in the home-living network (*r* = 0.229) and negative in the institutional-living network (*r* = −0.123) (Figure 3). Thus, the observed difference in overall network structure appeared to be concentrated primarily in this single edge rather than reflecting widespread differences across all domain relationships.

### 3.4 Statistical indirect associations involving living arrangement, sensory perception and social participation, and profile membership

An exploratory statistical indirect-association analysis was conducted to examine whether sensory perception and social participation were involved in the statistical association between living arrangement and functional impairment profile membership. Living arrangement was coded as 0 for institutional living and 1 for home living. Sensory perception and social participation was entered as a continuous intermediate variable, and the severe multidomain impairment profile was specified as the reference outcome category. The models were adjusted for sex, age, current marital status, chronic disease status, and medical insurance type. Because all variables were measured concurrently, the results were interpreted as statistical indirect associations rather than causal mediation effects.

After adjustment for the specified covariates, home living was associated with a higher sensory perception and social participation score than institutional living (*B* = 1.595, 95% CI: 1.108–2.083, *P* < 0.001). With the severe multidomain impairment profile as the reference category, sensory perception and social participation was not significantly associated with membership in the impaired mental state with relatively preserved physical function profile (*B* = 0.117, *P* = 0.261). Higher scores were positively associated with membership in the moderate functional impairment profile (*B* = 0.438, *P* < 0.001) and the relatively preserved function profile (*B* = 1.634, *P* < 0.001).

The bootstrap statistical indirect association was not significant for the impaired mental state with relatively preserved physical function profile vs. the severe multidomain impairment profile (estimate = 0.186, 95% bootstrap CI: −0.187 to 0.616). Positive statistical indirect associations were observed for the moderate functional impairment profile (estimate = 0.699, 95% bootstrap CI: 0.412–1.113) and the relatively preserved function profile (estimate = 2.607, 95% bootstrap CI: 1.751–5.366).

**Figure 4:**
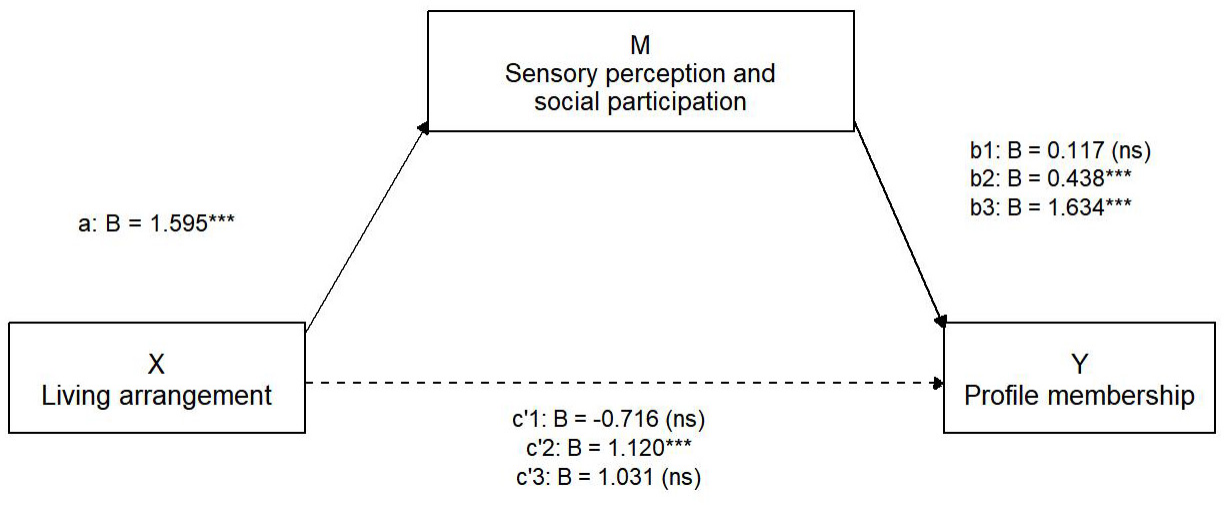
Statistical indirect association model linking living arrangement, sensory perception and social participation, and functional impairment profile membership. *X* = living arrangement (0 = institutional living, 1 = home living); *M* = sensory perception and social participation score; *Y* = functional impairment profile membership, with Profile 1 (severe multidomain impairment) as the reference category. b1 and c1′ represent Profile 2 vs. Profile 1; b2 and c2′ represent Profile 3 vs. Profile 1; and b3 and c3′ represent Profile 4 vs. Profile 1. Values are unstandardized regression coefficients. The models were adjusted for sex, age, current marital status, chronic disease status, and medical insurance type. ***P < 0.001; ns, not statistically significant. Given the cross-sectional design, the model describes statistical indirect associations and does not establish temporal ordering or causality.

For the moderate functional impairment profile, both the direct association (estimate = 1.120, 95% bootstrap CI: 0.505–1.883) and the statistical indirect association were statistically significant. For the relatively preserved function profile, the statistical indirect association was significant, whereas the direct association was not (estimate = 1.031, 95% bootstrap CI: −1.115 to 2.905). Total associations were observed for the moderate functional impairment profile (estimate = 1.510, 95% bootstrap CI: 0.939–2.236) and the relatively preserved function profile (estimate = 2.090, 95% bootstrap CI: 1.013–3.629), but not for the impaired mental state with relatively preserved physical function profile. Because the multinomial outcome model was non-linear, the direct, indirect, and total coefficients were not interpreted as an exact additive decomposition (Table 4; Figure 4).

**Table 4:** Statistical indirect associations through sensory perception and social participation between living arrangement and functional impairment profile membership.

Outcome comparison	Association	Estimate	Bootstrap 95% CI	Interpretation
Profile 2 vs. Profile 1	Statistical indirect association (a × b)	0.186	−0.187 to 0.616	Not significant
	Direct association	−0.716	−2.342 to 0.289	Not significant
	Total association	−0.627	−2.249 to 0.358	Not significant
Profile 3 vs. Profile 1	Statistical indirect association (a × b)	**0.699**	**0.412 to 1.113**	Significant
	Direct association	**1.120**	**0.505 to 1.883**	Significant
	Total association	**1.510**	**0.939 to 2.236**	Significant
Profile 4 vs. Profile 1	Statistical indirect association (a × b)	**2.607**	**1.751 to 5.366**	Significant
	Direct association	1.031	−1.115 to 2.905	Not significant
	Total association	**2.090**	**1.013 to 3.629**	Significant

Profile 1, the severe multidomain impairment profile, was used as the reference outcome category. Living arrangement was coded as 0 for institutional living and 1 for home living. The models were adjusted for sex, age, current marital status, chronic disease status, and medical insurance type. Estimates are unstandardized coefficients. Confidence intervals were obtained from 5,000 nonparametric bootstrap samples. A 95% confidence interval that did not include zero was considered statistically significant. Because multinomial logistic regression is non-linear, the direct association, statistical indirect association, and total association estimates should not be interpreted as an exact additive decomposition. The analysis used most likely profile assignment and did not account for classification uncertainty in the same manner as R3STEP. Given the cross-sectional design, these estimates describe statistical indirect associations and do not establish temporal ordering or causality. Bold values indicate statistically significant associations, defined as a 95% bootstrap confidence interval that does not include zero.

## 4 Discussion

This study identified four functional impairment profiles among older adults with disability managed within the same community health service catchment: severe multidomain impairment, impaired mental state with relatively preserved physical function, moderate functional impairment, and relatively preserved function. The findings extend assessment based on an overall disability grade by showing that similar levels of apparent dependency may arise from different combinations of physical and mental impairment. This is consistent with person-centered research identifying heterogeneous disability profiles and distinct symptom patterns among older adults rather than a single uniform severity continuum (15, 27). The broad profile structure was reproduced in the four-domain sensitivity analysis, which reduces concern that it resulted solely from excluding sensory perception and social participation from the primary LPA. The profiles describe current combinations of functioning; they are not established disease stages or prognostic categories.

The profiles may be most useful as prompts for further assessment rather than as automatic prescriptions for care. The moderate functional impairment profile, which included more than half of the sample, retained meaningful capacity across several domains. For this group, the management issue is how to provide necessary assistance while preserving activities that can still be performed safely. The severe multidomain impairment profile may warrant broader review of nursing dependency, mobility, nutrition, medical complexity, complication risk, and caregiver support. The relatively preserved profile may require monitoring and preventive support rather than intensive substitution of care. These interpretations should remain person-centered because institutional organization can influence residents' opportunities for engagement (28), social interventions depend on relational and implementation conditions (29), and care environments designed around individual preferences may support autonomy among residents with physical impairment (30). Thus, profile membership may indicate where assessment should be deepened, but it does not define a standardized care package.

The impaired mental state with relatively preserved physical function profile is particularly important for practice because an assessment focused mainly on activities of daily living could underestimate supervision, communication, and safety needs. Relatively preserved mobility may coexist with impaired orientation, comprehension, expression, mood, behavior, or judgment. Evidence from trials in residential dementia care suggests that personalized one-to-one and preference-based psychosocial activities can improve engagement, affect, or behavioral outcomes (31, 32). These studies do not validate a specific intervention for the profile identified here, but they support the broader principle that preserved physical capacity should not be equated with low care need. Individuals in this profile may require more detailed assessment of cognition, communication, sensory function, emotional symptoms, decision-making capacity, and environmental risk.

After expanded adjustment for demographic, socioeconomic, family-resource, and health-related variables, home living was associated with greater odds of membership in both the moderate functional impairment and relatively preserved function profiles rather than the severe multidomain impairment profile. The estimate for the relatively preserved function profile was imprecise because only 20 participants belonged to this profile. The living-arrangement estimates remained similar across the sequential R3STEP models, with no material attenuation or reversal after educational attainment, household income, medical insurance, number of children, chronic disease status, and geriatric syndrome burden were added.

This stability indicates that the measured covariates included in the expanded models did not explain the observed associations, but it should not be interpreted as evidence that confounding was eliminated. Educational attainment and household income were measured in broad categories, and number of children does not directly represent the availability, intensity, or quality of family caregiving. Selection into institutional care may also depend on pre-admission functional status, supervision requirements, family relationships, access to formal care, and the characteristics of available institutions. The findings therefore do not demonstrate that home living preserves function or that institutional living causes more severe impairment. Living arrangement should instead be interpreted as a marker of a broader care and selection context. Participation and access to community resources may form part of this broader context. Previous longitudinal and quasi-experimental studies have associated social participation and community-resource use with subsequent care needs or functional outcomes (33, 34). However, those studies involved different populations and designs and cannot determine the direction of association in the present cross-sectional sample. The current results therefore support further investigation of the care contexts associated with living arrangement, rather than comparison of home and institutional settings as inherently beneficial or harmful.

Sensory perception and social participation differed across the four profiles in the univariate analysis and contributed to the exploratory network and statistical indirect-association analyses, but it was not included in the primary fully adjusted R3STEP model. An exploratory R3STEP extension that added this domain produced unstable estimates for the smallest profile, consistent with sparse-data separation, and was therefore not interpreted. The present study consequently does not establish sensory perception and social participation as an independent, classification-adjusted correlate of profile membership. Nevertheless, low sensory perception and social participation scores may still have practical relevance because sensory limitations can affect communication, environmental orientation, confidence, and opportunities for participation. Previous research has linked sensory impairment with depressive symptoms partly through restrictions in daily activity and social participation (35). Studies in long-term care have also emphasized hearing and vision screening and the adaptation of communication support (36–38). Low scores in this domain may therefore justify more detailed sensory, communication, and participation assessment, but the present data do not show that modifying this domain would change profile membership.

The findings also indicate that social participation should not be reduced to attendance at organized activities. Residents describe engagement in terms of personal meaning, choice, familiarity, and the fit between activities and their abilities (39), and tailored activity programs may improve engagement or behavioral outcomes in selected populations (40). At the same time, adding multiple poorly coordinated interventions can increase treatment burden for people with multimorbidity (41), while perceived autonomy remains a central ethical and practical concern in residential care (42). Profile-informed planning should therefore emphasize a limited number of feasible, preference-concordant supports, preserve choice where possible, and avoid replacing retained abilities merely for administrative convenience.

The statistical indirect associations involving sensory perception and social participation should be interpreted as overlap among concurrently measured variables rather than as causal mediation. Retained physical and mental function may enable communication, travel, and participation, whereas better sensory access and participation opportunities may coexist with functional maintenance. Reverse and reciprocal relationships are equally plausible. Common-method bias is also possible because all four domains were derived from the same assessment instrument. Moreover, the statistical indirect-association analysis used most likely profile assignment and a more limited covariate set than the primary R3STEP model. Although the four-domain sensitivity analysis reproduced the general profile structure, it did not establish sensory perception and social participation as an independent mechanism. The expanded R3STEP findings for living arrangement therefore provide the primary classification-adjusted evidence, whereas the indirect-association analysis should be regarded as hypothesis-generating.

The present dataset and national aging cohorts provide different and complementary forms of evidence. National cohorts offer broader population coverage, longitudinal follow-up, and more extensive socioeconomic and family-context variables, whereas the present study provides contemporaneous domain-level assessments collected directly according to GB/T 42195–2022 within a common community health service catchment. Because commonly used national cohort waves were collected before implementation of the 2022 standard or used cohort-specific assessment instruments, they do not directly generate the four domain scores specified by GB/T 42195–2022. The value of the present data therefore lies in examining functional patterns under the national assessment framework, not in replacing the broader epidemiological strengths of national databases. From a service perspective, the identified profiles may complement the official GB/T 42195–2022 grade by providing a second descriptive layer. Overall grades remain necessary for eligibility determination, administrative management, and broad risk stratification. Profile information may additionally indicate whether further assessment should prioritize multidomain dependency, mental-state and safety concerns, preservation of retained capacity, or sensory and participation barriers. However, the present study did not test whether adding profile information improves coordination, efficiency, care quality, or patient outcomes.

Long-term care residents may follow different trajectories of functional decline over time (43), and ongoing efforts to integrate medical, community, home-based, and institutional care in China continue to require coordination across service boundaries (44). The observed heterogeneity also aligns with broader person-centered evidence. Studies have identified distinct social-participation profiles among community-dwelling older adults (45), joint patterns of living arrangement and participation associated with mental health (46), and demoralization profiles related to active aging among older adults with disability (47). Other longitudinal and long-term care research has linked participation patterns with subsequent cognitive function and social networks with cognition and quality of life (48, 49). These studies support examining the quality and accessibility of social relationships rather than activity frequency alone. They also provide context for the present association between sensory perception and social participation and profile membership, but they do not establish that increasing participation would move an individual from one profile to another.

The exploratory network analysis provided a supplementary description of relationships among the four functional domains. Self-care ability and basic mobility, and mental state and sensory perception and social participation, showed comparatively strong conditional associations in the full sample. Evidence from randomized trials indicates that supervised exercise can improve aspects of physical function among older adults in residential care (50), which supports continued attention to mobility maintenance but does not identify mobility as a causal network target. Multidimensional network research in community-dwelling older adults likewise shows that cognitive, physical, and quality-of-life domains can be interrelated (51). However, the network contained only four aggregate nodes, centrality stability was inadequate in the home-living subgroup, and only the self-care ability–mental state edge differed between living-arrangement groups after correction for multiple comparisons. The significant overall structure test should therefore not be interpreted as evidence of widespread differences across all domain relationships. Centrality rankings and individual edges should not be used to designate causal mechanisms or intervention priorities.

The principal practical contribution of this study is therefore a testable framework for differentiated assessment rather than evidence for immediate redistribution of services or resources. Community health service centers could pilot the use of profile information alongside the official grade to support multidisciplinary review, identify potentially overlooked mental or sensory needs, and facilitate handoffs between home and institutional services. Such an approach should be evaluated prospectively against usual grading alone. Relevant outcomes would include assessment agreement, referral appropriateness, functional change, adverse events, quality of life, caregiver burden, service use, staffing requirements, and cost-effectiveness. Without such evidence, claims that profile-based management improves resource allocation or care quality would be premature.

Finally, the policy implications should be confined to the present study setting. Because participants were recruited from a single community health service catchment, the observed profile proportions should not be interpreted as population estimates for Kunming, Yunnan Province, or China. Until the findings are replicated in multicenter and longitudinal studies, they are most appropriately used for service-level hypothesis generation rather than broad policy recommendations.

## 5 Strengths and limitations

This study used a person-centered approach to identify heterogeneity in functional impairment among older adults with disability assessed under a common national standard. Profile selection jointly considered model fit, classification quality, class size, parsimony, and substantive interpretability, and R3STEP was used to account for uncertainty in latent profile classification. The primary R3STEP model incorporated measured demographic, socioeconomic, family-resource, and health-related variables, including educational attainment, household income, number of children, medical insurance, chronic disease status, and geriatric syndrome burden. Participant recruitment and non-inclusion were reported transparently, and the primary findings were supplemented by competing-profile comparisons, a four-domain sensitivity analysis, exploratory network analysis, and statistical indirect-association analysis.

Several limitations should be acknowledged. First, the cross-sectional design precluded causal or temporal inference. Second, purposive sampling from a single community health service catchment and the exclusion or non-participation of some eligible individuals may have introduced selection bias and limited generalizability. Third, although the expanded R3STEP model adjusted for demographic, socioeconomic, family-resource, and health-related variables, living arrangement was not randomly assigned, and residual confounding related to pre-existing disability, actual family support, reasons for institutional admission, length of stay, and institutional care characteristics could not be excluded. Fourth, the relatively preserved function profile contained only 20 participants, resulting in imprecise estimates. Finally, the statistical indirect-association analysis used most likely profile assignment and a more limited covariate set than the primary R3STEP model, while the home-living network showed inadequate centrality stability; these supplementary findings should therefore be considered exploratory.

## 6 Conclusions

Functional impairment among older adults with disability managed within a community health service catchment was heterogeneous, with four profiles identified: severe multidomain impairment, impaired mental state with relatively preserved physical function, moderate functional impairment, and relatively preserved function. After adjustment for measured demographic, socioeconomic, family-resource, and health-related variables and uncertainty in profile classification, home living was associated with membership in the moderate functional impairment and relatively preserved function profiles rather than the severe multidomain impairment profile. However, the estimate for the smallest profile was imprecise, and the cross-sectional design, care-selection processes, and residual confounding preclude causal interpretation. Findings involving sensory perception and social participation from the descriptive, network, and statistical indirect-association analyses were exploratory and did not establish an independent classification-adjusted association or causal mechanism. Profile-based assessment may provide a complementary descriptive layer alongside the official GB/T 42195–2022 grade and may help identify areas requiring further functional assessment. Its value for care coordination, service allocation, and patient outcomes requires confirmation in multicenter longitudinal and prospective implementation studies.

## Data Availability

The datasets generated and analyzed during the current study are not publicly available because they contain potentially sensitive health information and are subject to confidentiality requirements. De-identified data may be made available by the corresponding authors upon reasonable request and subject to applicable ethical and institutional requirements.
