# Correction: Functional impairment profiles and long-term care implications among community health service-managed older adults with disability in China: a latent profile and network analysis study

**DOI:** 10.3389/fpubh.2026.2024249

**Published:** 2026-10-29

**Authors:** Ruishan Liu, Yiheng Chen, Ting Niu, Dingyi Zhang, Yuelin Li, Dan Yang, Yulin Lu

**Affiliations:** 1 School of Nursing, Kunming Medical University, Kunming, China; 2 Jinma Subdistrict Health Service Center, Guandu District, Kunming, China

**Keywords:** functional impairment, latent profile analysis, living arrangement, long-term care, network analysis, older adults with disability

In the published article, the order of the authors in the author list was incorrect.

The author list was previously:

“Ruishan Liu, Yiheng Chen, Ting Niu, Dingyi Zhang, Yuelin Li, Yulin Lu and Dan Yang”

The correct order of the authors is:

“Ruishan Liu, Yiheng Chen, Ting Niu, Dingyi Zhang, Yuelin Li, Dan Yang and Yulin Lu”

The original version of this article has been updated.

