# Supplementary material for: Functional impairment profiles and long-term care implications among community health service-managed older adults with disability in China: a latent profile and network analysis study
Source: Front Public Health. 2026 Aug 31;14:1885713. doi: 10.3389/fpubh.2026.1885713 (PMC13572153; doi:10.3389/fpubh.2026.1885713)
Supplement: Supplementary file 1 [file Table_1.docx]

**Supplementary Figure S1. Comparison of the estimated functional impairment profiles in the three-profile and four-profile solutions.**

**Note:** Panel A presents the three-profile solution, and Panel B presents the four-profile solution. Domain scores were linearly transformed to a 0–100 scale, with higher scores indicating better functional ability. Points and lines represent model-estimated class-specific means obtained from the Mplus latent profile models. Class sizes were determined according to most likely latent profile membership.
