# Supplementary material for: Functional impairment profiles and long-term care implications among community health service-managed older adults with disability in China: a latent profile and network analysis study
Source: Front Public Health. 2026 Aug 31;14:1885713. doi: 10.3389/fpubh.2026.1885713 (PMC13572153; doi:10.3389/fpubh.2026.1885713)
Supplement: Supplementary file 2 [file Table_2.docx]

**Supplementary Table S1. Class sizes for competing latent profile models**

| **Model** | **Latent class** | **Class size, n** | **Class proportion, %** |
| --- | --- | --- | --- |
| 1-profile | Class 1 | 361 | 100.0 |
| 2-profile | Class 1 | 122 | 33.8 |
|  | Class 2 | 239 | 66.2 |
| **3-profile** | **Class 1** | **115** | **31.9** |
|  | **Class 2** | **43** | **11.9** |
|  | **Class 3** | **203** | **56.2** |
| **4-profile** | **Class 1** | **102** | **28.3** |
|  | **Class 2** | **46** | **12.7** |
|  | **Class 3** | **193** | **53.5** |
|  | **Class 4** | **20** | **5.5** |
| 5-profile | Class 1 | 16 | 4.4 |
|  | Class 2 | 185 | 51.2 |
|  | Class 3 | 104 | 28.8 |
|  | Class 4 | 35 | 9.7 |
|  | Class 5 | 21 | 5.8 |

**Note:** Class sizes and proportions were based on participants’ most likely latent class membership. Percentages may not total exactly 100.0% because of rounding. Latent class numbers are model-specific and do not necessarily represent equivalent profiles across models. The four-profile solution was selected as the final model.

**Supplementary Table S2. Model fit, class-specific estimated means, and classification quality of the competing three-profile and four-profile solutions based on the three core functional domains**

**Panel A：Model fit indices**

| **Model** | **Log-likelihood** | **Free parameters** | **AIC** | **BIC** | **aBIC** | **Entropy** | **Adjusted** | |
| --- | --- | --- | --- | --- | --- | --- | --- | --- |
|  |  |  |  |  |  |  | **LMRT P** | **BLRT P** |
| Three-profile | −4876.661 | 14 | 9781.321 | 9835.766 | 9791.350 | 0.847 | 0.0489 | <0.001 |
| Four-profile | −4848.384 | 18 | 9732.768 | 9802.768 | 9745.662 | 0.859 | <0.001 | <0.001 |

**Panel B：Class-specific model-estimated means**

| **Model** | **Profile** | **Most likely n (%)** | **Self-care ability, mean (SE)** | **Basic mobility, mean (SE)** | **Mental state, mean (SE)** |
| --- | --- | --- | --- | --- | --- |
| Three-profile solution | Severe multidomain impairment | 115 (31.9%) | 26.298 (2.060) | 21.284 (2.724) | 32.598 (1.940) |
|  | Impaired mental state with relatively preserved physical function | 43 (11.9%) | 94.773 (1.443) | 78.353 (11.810) | 9.012 (2.224) |
|  | Moderate functional impairment | 203 (56.2%) | 62.257 (1.323) | 55.817 (2.082) | 45.694 (2.190) |
| Four-profile solution | Severe multidomain impairment | 102 (28.3%) | 24.352 (1.980) | 19.667 (2.394) | 32.600 (2.176) |
|  | Impaired mental state with relatively preserved physical function | 46 (12.7%) | 94.388 (1.317) | 76.345 (7.218) | 8.923 (1.952) |
|  | Moderate functional impairment | 193 (53.5%) | 59.093 (1.423) | 52.171 (1.835) | 41.806 (1.531) |
|  | Relatively preserved function | 20 (5.5%) | 77.724 (2.532) | 79.052 (4.840) | 76.747 (4.675) |

**Note:**Model-estimated residual variances were constrained to be equal across profiles, and within-profile covariances were fixed at zero.

**Panel C：Average posterior probability matrices**

| **Model** | **Most likely profile** | **Profile 1** | **Profile 2** | **Profile 3** | **Profile 4** |
| --- | --- | --- | --- | --- | --- |
| Three-profile solution | Profile 1 | **0.910** | 0.000 | 0.090 |  |
|  | Profile 2 | 0.000 | **0.924** | 0.076 |  |
|  | Profile 3 | 0.039 | 0.017 | **0.944** |  |
| Four-profile solution | Profile 1 | **0.914** | 0.000 | 0.086 | 0.000 |
|  | Profile 2 | 0.000 | **0.945** | 0.053 | 0.002 |
|  | Profile 3 | 0.041 | 0.010 | **0.926** | 0.024 |
|  | Profile 4 | 0.000 | 0.004 | 0.124 | **0.872** |

**Note:** AIC, Akaike information criterion; BIC, Bayesian information criterion; aBIC, sample size-adjusted Bayesian information criterion; LMRT, Lo–Mendell–Rubin adjusted likelihood ratio test; BLRT, bootstrap likelihood ratio test; SE, standard error. Class sizes were based on most likely latent profile membership. Profile means and standard errors were estimated by the latent profile models. Rows in the posterior probability matrices represent most likely profile membership, and columns represent estimated posterior probabilities. The four-profile solution was retained after consideration of model fit, classification quality, class size, parsimony, and substantive interpretability.

**Supplementary Table S3. Cross-classification and agreement of most likely profile membership between the three-profile and four-profile solutions**

**Panel A. Cross-classification**

| **Three-profile solution** | **Severe multidomain impairment** | **Impaired mental state with relatively preserved physical function** | **Moderate functional impairment** | **Relatively preserved function** | **Total** |
| --- | --- | --- | --- | --- | --- |
| Severe multidomain impairment | 102 (88.7%) | 0 (0.0%) | 13 (11.3%) | 0 (0.0%) | 115 |
| Impaired mental state with relatively preserved physical function | 0 (0.0%) | 43 (100.0%) | 0 (0.0%) | 0 (0.0%) | 43 |
| Moderate functional impairment | 0 (0.0%) | 3 (1.5%) | 180 (88.7%) | 20 (9.9%) | 203 |
| **Total** | **102** | **46** | **193** | **20** | **361** |

**Panel B. Classification agreement**

| **Agreement measure** | **Estimate** |
| --- | --- |
| Adjusted Rand index | 0.746 |
| Overall agreement after collapsing Profiles 3 and 4 of the four-profile solution | 95.6% |
| Cohen’s kappa after collapsing Profiles 3 and 4 | 0.921 |

**Note:** Values in Panel A are presented as n (row percentage). Profile labels were aligned according to the class-specific patterns of self-care ability, basic mobility, and mental state rather than the original numerical class labels generated by Mplus. The adjusted Rand index was used to assess overall similarity between the three-profile and four-profile classifications. Because the relatively preserved function profile in the four-profile solution was primarily differentiated from the broader moderate functional impairment profile in the three-profile solution, these two four-profile categories were combined only for the calculation of sensitivity agreement and Cohen’s kappa. The four-profile solution remained the primary analytical model.

**Supplementary Table S4. Fit indices and classification quality of the one- to five-profile solutions including all four functional domains**

| **Profile** | **Log-likelihood** | **Free parameters** | **AIC** | **BIC** | **aBIC** | **Entropy** | ***P*** | | **Most likely class sizes, n (%)** | **Diagonal average posterior probabilities** |
| --- | --- | --- | --- | --- | --- | --- | --- | --- | --- | --- |
|  |  |  |  |  |  |  | **LMRT** | **BLRT** |  |  |
| 1 | −6531.735 | 8 | 13079.470 | 13110.581 | 13085.201 | — | — | — | 361 (100.0%) | 1.000 |
| 2 | −6424.802 | 13 | 12875.604 | 12926.159 | 12884.917 | 0.716 | <0.001 | <0.001 | 153 (42.4%)/208 (57.6%) | 0.890/0.933 |
| 3 | −6339.052 | 18 | 12714.105 | 12784.105 | 12726.999 | 0.850 | 0.0006 | <0.001 | 193 (53.5%)/123 (34.1%)/45 (12.5%) | 0.932/0.932/0.946 |
| **4** | **−6278.968** | **23** | **12603.935** | **12693.379** | **12620.411** | **0.843** | **0.1562** | **<0.001** | **104 (28.8%)/46 (12.7%)/178 (49.3%)/33 (9.1%)** | **0.916/0.951/0.900/0.901** |
| 5 | −6238.303 | 28 | 12532.607 | 12641.495 | 12552.664 | 0.876 | 0.0001 | <0.001 | 103 (28.5%)/172 (47.6%)/17 (4.7%)/34 (9.4%)/35 (9.7%) | 0.941/0.910/0.907/0.892/0.943 |

**Note:** AIC, Akaike information criterion; BIC, Bayesian information criterion; aBIC, sample size-adjusted Bayesian information criterion; LMRT, Lo–Mendell–Rubin adjusted likelihood ratio test; BLRT, bootstrap likelihood ratio test. The four-domain models included self-care ability, basic mobility, mental state, and sensory perception and social participation, with all domain scores linearly transformed to a 0–100 scale and higher scores indicating better functional ability. Class sizes and proportions were based on most likely latent profile membership. The four-profile solution was retained for sensitivity analysis after considering information criteria, classification quality, class size, parsimony, and substantive interpretability. Although the adjusted LMRT comparing the four-profile with the three-profile solution was not statistically significant, the four-profile solution yielded an interpretable additional relatively preserved function profile without producing a class smaller than 5%. The five-profile solution generated a small class comprising 4.7% of the sample and provided limited additional substantive clarity.

**Supplementary Table S5. Class-specific estimates and classification quality of the retained four-profile solution including all four functional domains**

**Panel A. Class sizes and model-estimated domain means**

| **Profile** | **Most likely n (%)** | **Self-care ability, mean (SE)** | **Basic mobility, mean (SE)** | **Mental state, mean (SE)** | **Sensory perception and social participation, mean (SE)** |
| --- | --- | --- | --- | --- | --- |
| Severe multidomain impairment | 104 (28.8%) | 24.823 (2.525) | 19.373 (2.740) | 31.946 (2.192) | 28.267 (1.261) |
| Impaired mental state with relatively preserved physical function | 46 (12.7%) | 94.123 (1.361) | 73.930 (6.173) | 8.704 (1.999) | 28.915 (1.864) |
| Moderate functional impairment | 178 (49.3%) | 58.125 (2.682) | 51.479 (3.555) | 41.187 (2.974) | 40.298 (3.709) |
| Relatively preserved function | 33 (9.1%) | 73.090 (6.281) | 74.181 (8.994) | 66.756 (12.382) | 64.790 (6.250) |

**Panel B. Average posterior probability matrix**

| **Most likely profile** | **Severe multidomain impairment** | **Impaired mental state with relatively preserved physical function** | **Moderate functional impairment** | **Relatively preserved function** |
| --- | --- | --- | --- | --- |
| Severe multidomain impairment | **0.916** | 0.000 | 0.084 | 0.000 |
| Impaired mental state with relatively preserved physical function | 0.000 | **0.951** | 0.047 | 0.002 |
| Moderate functional impairment | 0.037 | 0.010 | **0.900** | 0.053 |
| Relatively preserved function | 0.000 | 0.010 | 0.089 | **0.901** |

**Note:** Domain scores were linearly transformed to a 0–100 scale, with higher scores indicating better functional ability. Class sizes were based on most likely latent profile membership. Means and standard errors were model-estimated values. Rows in Panel B represent most likely profile membership, and columns represent estimated posterior probabilities. Model-estimated residual variances were constrained to be equal across profiles, and within-profile covariances were fixed at zero.

**Supplementary Table S6. Cross-classification and agreement of profile membership between the primary three-domain model and the four-domain sensitivity model**

**Panel A. Cross-classification of most likely profile membership**

| **Primary three-domain model** | **Severe multidomain impairment** | **Impaired mental state with relatively preserved physical function** | **Moderate functional impairment** | **Relatively preserved function** | **Total** |
| --- | --- | --- | --- | --- | --- |
| Severe multidomain impairment | 97 (95.1%) | 0 (0.0%) | 5 (4.9%) | 0 (0.0%) | 102 |
| Impaired mental state with relatively preserved physical function | 0 (0.0%) | 44 (95.7%) | 0 (0.0%) | 2 (4.3%) | 46 |
| Moderate functional impairment | 7 (3.6%) | 2 (1.0%) | 173 (89.6%) | 11 (5.7%) | 193 |
| Relatively preserved function | 0 (0.0%) | 0 (0.0%) | 0 (0.0%) | 20 (100.0%) | 20 |
| **Total** | **104** | **46** | **178** | **33** | **361** |

**Panel B. Classification agreement between the two four-profile solutions**

| Measure | Estimate |
| --- | --- |
| Adjusted Rand index | 0.799 |
| Overall classification agreement | 92.5% |
| Cohen's kappa | 0.882 |

**Supplementary Table S7. Participant characteristics and functional impairment profile distribution according to living arrangement**

| **Variable** | **Institutional living (n = 225)** | **Home living with family (n = 111)** | **Living alone (n = 25)** | **Test statistic** | **P value** |
| --- | --- | --- | --- | --- | --- |
| **Sex** |  |  |  |  |  |
| Male | 87 (38.7%) | 57 (51.4%) | 9 (36.0%) | 5.346ᵃ | 0.069 |
| Female | 138 (61.3%) | 54 (48.6%) | 16 (64.0%) |  |  |
| **Age, years** |  |  |  |  |  |
| <70 | 18 (8.0%) | 10 (9.0%) | 0 (0.0%) | 12.322ᵃ | 0.055 |
| 70–79 | 60 (26.7%) | 42 (37.8%) | 12 (48.0%) |  |  |
| 80–89 | 89 (39.6%) | 43 (38.7%) | 9 (36.0%) |  |  |
| ≥90 | 58 (25.8%) | 16 (14.4%) | 4 (16.0%) |  |  |
| **Marital status** |  |  |  |  |  |
| Never married | 8 (3.6%) | 1 (0.9%) | 0 (0.0%) | 33.453ᵇ | <0.001 |
| Married | 85 (37.8%) | 75 (67.6%) | 12 (48.0%) |  |  |
| Divorced | 12 (5.3%) | 0 (0.0%) | 3 (12.0%) |  |  |
| Widowed | 120 (53.3%) | 35 (31.5%) | 10 (40.0%) |  |  |
| **Educational attainment** |  |  |  |  |  |
| No formal schooling | 68 (30.2%) | 29 (26.1%) | 4 (16.0%) | 10.968ᵇ | 0.183 |
| Primary school | 63 (28.0%) | 37 (33.3%) | 10 (40.0%) |  |  |
| Junior high school | 58 (25.8%) | 29 (26.1%) | 5 (20.0%) |  |  |
| Senior high school/technical secondary school | 24 (10.7%) | 15 (13.5%) | 6 (24.0%) |  |  |
| College or above | 12 (5.3%) | 1 (0.9%) | 0 (0.0%) |  |  |
| **Monthly household income, RMB** |  |  |  |  |  |
| <3,000 | 87 (38.7%) | 43 (38.7%) | 7 (28.0%) | 7.375ᵃ | 0.288 |
| 3,000–5,999 | 106 (47.1%) | 47 (42.3%) | 11 (44.0%) |  |  |
| 6,000–9,999 | 23 (10.2%) | 13 (11.7%) | 3 (12.0%) |  |  |
| ≥10,000 | 9 (4.0%) | 8 (7.2%) | 4 (16.0%) |  |  |
| **Number of children** |  |  |  |  |  |
| 0 | 18 (8.0%) | 13 (11.7%) | 3 (12.0%) | 3.428ᵃ | 0.754 |
| 1 | 71 (31.6%) | 32 (28.8%) | 6 (24.0%) |  |  |
| 2 | 89 (39.6%) | 42 (37.8%) | 8 (32.0%) |  |  |
| ≥3 | 47 (20.9%) | 24 (21.6%) | 8 (32.0%) |  |  |
| **Primary caregiver** |  |  |  |  |  |
| Spouse | 0 (0.0%) | 39 (35.1%) | 7 (28.0%) | 379.837ᵇ | <0.001 |
| Children or children-in-law | 12 (5.3%) | 54 (48.6%) | 14 (56.0%) |  |  |
| Other relatives | 0 (0.0%) | 12 (10.8%) | 2 (8.0%) |  |  |
| Institutional care worker | 208 (92.4%) | 0 (0.0%) | 0 (0.0%) |  |  |
| No regular caregiver | 5 (2.2%) | 6 (5.4%) | 2 (8.0%) |  |  |
| **Medical insurance type** |  |  |  |  |  |
| Employee medical insurance | 114 (50.7%) | 53 (47.7%) | 12 (48.0%) | 2.737ᵃ | 0.603 |
| Urban–rural resident medical insurance | 72 (32.0%) | 44 (39.6%) | 8 (32.0%) |  |  |
| No medical insurance | 39 (17.3%) | 14 (12.6%) | 5 (20.0%) |  |  |
| **Geriatric syndrome count** |  |  |  |  |  |
| 0 | 20 (8.9%) | 22 (19.8%) | 7 (28.0%) | 15.018ᵃ | 0.005 |
| 1–2 | 154 (68.4%) | 62 (55.9%) | 16 (64.0%) |  |  |
| ≥3 | 51 (22.7%) | 27 (24.3%) | 2 (8.0%) |  |  |
| **Chronic disease** |  |  |  |  |  |
| Yes | 147 (65.3%) | 72 (64.9%) | 16 (64.0%) | 0.021ᵃ | 0.989 |
| No | 78 (34.7%) | 39 (35.1%) | 9 (36.0%) |  |  |
| **Functional impairment profile** |  |  |  |  |  |
| Profile 1: Severe multidomain impairment | 84 (37.3%) | 12 (10.8%) | 6 (24.0%) | 60.616ᵃ | <0.001 |
| Profile 2: Impaired mental state with relatively preserved physical function | 41 (18.2%) | 4 (3.6%) | 1 (4.0%) |  |  |
| Profile 3: Moderate functional impairment | 93 (41.3%) | 82 (73.9%) | 18 (72.0%) |  |  |
| Profile 4: Relatively preserved function | 7 (3.1%) | 13 (11.7%) | 0 (0.0%) |  |  |

**Note:** Data are presented as n (column %). Percentages describe the distribution within each living-arrangement group. ᵃ Pearson’s chi-square test. ᵇ Fisher–Freeman–Halton exact test with P values estimated using 10,000 Monte Carlo samples. All tests were two-sided, and P<0.05 was considered statistically significant. Primary caregiver was reported descriptively because it was structurally related to living arrangement and was not treated as a conventional covariate in the primary multivariable model. The differences in profile distribution represent unadjusted sample associations and should not be interpreted as effects of living arrangement.

**Supplementary Table S8. Association between living arrangement and functional impairment profile membership across sequential R3STEP models**

| **Outcome comparison** | **Model 0, OR (95% CI)** | **P value** | **Model 1, aOR (95% CI)** | **P value** | **Model 2, aOR (95% CI)** | **P value** |
| --- | --- | --- | --- | --- | --- | --- |
| Profile 2 vs Profile 1 | 0.546 (0.133–2.247) | 0.402 | 0.576 (0.138–2.407) | 0.450 | 0.570 (0.131–2.476) | 0.453 |
| Profile 3 vs Profile 1 | 6.918 (3.191–15.000) | <0.001 | 6.858 (2.832–16.606) | <0.001 | 7.046 (2.827–17.562) | <0.001 |
| Profile 4 vs Profile 1 | 12.111 (3.397–43.177) | <0.001 | 14.081 (3.111–63.739) | 0.001 | 15.408 (2.652–89.528) | 0.002 |

**Note:** Model 0 included living arrangement only. Model 1 adjusted for sex, age, current marital status, educational attainment, monthly household income, and medical insurance type. Model 2 additionally adjusted for number of children, chronic disease status, and geriatric syndrome burden. Profile 1, the severe multidomain impairment profile, was the reference outcome category. OR, odds ratio; aOR, adjusted odds ratio; CI, confidence interval.
